# Consumption of Maternal Placenta in Humans and Nonhuman Mammals: Beneficial and Adverse Effects

**DOI:** 10.3390/ani10122398

**Published:** 2020-12-15

**Authors:** Daniel Mota-Rojas, Agustín Orihuela, Ana Strappini, Dina Villanueva-García, Fabio Napolitano, Patricia Mora-Medina, Hugo B. Barrios-García, Yuridia Herrera, Eunice Lavalle, Julio Martínez-Burnes

**Affiliations:** 1 Neurophysiology, Behavior and Animal Welfare Assessment, DPAA, Universidad Autónoma Metropolitana (UAM), 04960 Mexico City, Mexico; dmota100@yahoo.com.mx (D.M.-R.); herrerajannotta@gmail.com (Y.H.); amira.lavalle@gmail.com (E.L.); 2 Facultad de Ciencias Agropecuarias, Universidad Autónoma del Estado de Morelos, Cuernavaca, 62209 Morelos, Mexico; aorihuela@uaem.mx; 3 Animal Science Institute, Faculty of Veterinary Sciences, Universidad Austral de Chile, 5110566 Valdivia, Chile; anastrappini@uach.cl; 4 Division of Neonatology, National Institute of Health Hospital Infantil de México Federico Gómez, 06720 Mexico City, Mexico; dinavg21@yahoo.com; 5 Scuola di Scienze Agrarie, Forestali, Alimentari ed Ambientali, Università degli Studi della Basilicata, 85100 Potenza, Italy; fabio.napolitano@unibas.it; 6 Livestock Science Department, Facultad de Estudios Superiores Cuautitlán, Universidad Nacional Autónoma de México (UNAM), 54714 Mexico City, Mexico; mormed2001@yahoo.com.mx; 7 Graduate and Research Department, Faculty of Veterinary Medicine, Universidad Autónoma de Tamaulipas, Ciudad Victoria, 87000 Tamaulipas, Mexico; hbarrios@docentes.uat.edu.mx

**Keywords:** placenta, amniotic fluid, placentophagia, POEF, mammals, parturition

## Abstract

**Simple Summary:**

Placentophagia is the ingestion of the placenta and afterbirth components released during and after parturition. The maternal behavior is widespread in mammalians and takes place in nonhuman primates too. It can occur between related and unrelated female primates and female and male weanling siblings in rats and hamsters. There are reports of human placentophagia in North America where women consume their placenta, whether raw, cooked, dehydrated, processed, or encapsulated, assuming potential health benefits for mothers and their offspring. However, there are also possible detrimental health risks associated with placental consumption in women. There is no scientific research regarding the beneficial effects of human placentophagia, and available information is mostly hearsay. We discuss the cause-effect of placentophagy and the endocrine, nutritional, and analgesic proposed benefits and adverse effects of this practice that have been published in humans and nonhuman mammals.

**Abstract:**

Placentophagia is a common mammalian behavior, and the first scientific study of the potential effects of human maternal placentophagia on lactation was in 1917. More recently, in the 1970s, human placentophagia was reported in North America with a trend toward increased consumption. There are different hypotheses about the women and nonhuman mammals’ motivation towards placentophagia, but few have been subject to hypotheses testing. In women, the controversy continues; on the one hand, researchers attribute benefits like increased breast milk, weight gain in newborns, decreased postpartum depression and fatigue, and improved mothers’ mood. In contrast, bacterial or viral infections, hormonal, or trace elements that could become toxic for both the mother and baby are reported as possible health risks. Other reports argue a lack of scientific rigor to support the self-reported benefits of placentophagia. Also, the way the placenta is prepared (raw, cooked, dehydrated, processed, or encapsulated) alters its components, and thus the desired effects. This review provides relevant information and the different hypotheses and points of view around placentophagia. However, there are still questions to be resolved, and more studies are needed to confirm or reject the data generated so far about placentophagia in humans and nonhuman mammals.

## 1. Introduction

The placenta is a complex, temporal, and dynamic organ that performs various synthetic, secretory, filtering, analytical, and transport functions [1]. Placental tissue derives from trophoblasts, which develop from the same blastocyst as the embryo [2]. In mammals, it is the organ through which gases, nutrients, and respiratory wastes are exchanged between mother and fetus [3]. It has immunological importance facilitating embryonic and fetal development, as well as embryo survival. Furthermore, it contains stem cells that can be used for medical purposes, such as allografts [2,4].

A few seconds or hours after parturition, the placenta, amniotic fluid, and associated membranes are expelled. Placentophagia is a behavior present in almost all female terrestrial eutherian mammals (more than 4000 species), consisting of the ingestion of some or all of the placental components released during parturition [1,5,6]. However, this behavior does not occur in large aquatic (cetacean) or semi-aquatic (pinniped) mammals [7,8] and has not been documented in camelids [1].

Placentophagia has been thoroughly studied, primarily in mice (*Peromyscus californicus*) [9], hamsters (*Phodopus campbelli*) [10], rats (*Mus musculus*) [11], rabbits (*Oryctolagus cuniculus*) [12,13], ungulates [14,15], and carnivores [16], and regularly occurs in all nonhuman primate species [17,18,19]. In contrast, it is assumed that traditionally, women did not carry out this practice for socio-cultural reasons. Nevertheless, there is evidence that dried human placenta is a centuries-old Traditional Chinese Medicine remedy consumed by postpartum mothers [19]. However, dried human placenta is a remedy prescribed for various conditions, for both men and women, including chronic cough and male sexual dysfunction.

The first scientific study of human maternal placentophagia was conducted in 1917, in which the increase in protein and lactose in the milk of lactating women who consumed their dry placenta stood out [20]. More recently, the first consistent reports of human placentophagia originated from North America in the 1970s and spread out among women living in industrialized cities [21], leading to a trend for an increase in consumption of the placenta, whether raw, steeped in liquid, steamed, cooked, dehydrated, processed and encapsulated, or as a tea [22].

On the other hand, in some animals, the appetite for placental materials depends on the female’s reproductive stage, increasing during the peripartum period [12]. However, placentophagia is not exclusive to females, as some bi-parental and monogamous male rodents (mice, rats, and hamsters) also practice it [9,10,11,23]. Placentophagia is considered to have advantages not only for females but also for their offspring, such as increased mother–offspring interaction, potentiation of the action of brain opioids that promote caring behavior towards the young, aiding neonatal respiration by removing membranes from the nostrils, and providing analgesia enhancement in the parturient female [5]. In addition, it reduces the incidence of postpartum pseudopregnancy [5], prevents postpartum hemorrhages, decreases postpartum depression [24], promotes lactogenesis, and provides nutritional benefits [25]. In humans, placentophagia has been considered a puerperal remedy [22]; however, some studies indicate that human placenta does not provide all its benefits [26].

In light of these facts, this review aims to describe the causes and effects of placentophagia, the endocrine aspects involved, nutritional or analgesic benefits, and the adverse effects that have been published in humans and nonhuman mammals.

## 2. Factors that Explain the Phenomenon

There are different hypotheses about the motivation towards placentophagia, but not all have been proven. Kristal et al. [27] suggest that the causes that motivate placentophagia in animals depend on the taxonomic group to which they belong. This behavior would be part of the repertoire of innate behaviors that animals exhibit in the peripartum, such as nest construction or site displacement.

Other studies propose that the placental intake is due to the feeling of hunger after fasting during the peripartum. However, there is evidence that not all females stop eating before giving birth; for example, rats and giraffes continue to ingest food 24 h before delivery [1].

On the other hand, a temporary change in feeding preference during parturition may be why herbivorous animals practice this behavior. In this same sense, Kristal [1] mentions that some females (rats and monkeys) reject any meat after giving birth. Similar findings were found by Melo and González-Mariscal [12]: they offered rabbits (*Oryctolagus cuniculus*) liver and placenta during parturition and observed that the behavior of ingesting placenta increased in this state up to practically a 100% frequency. In contrast, liver consumption was only 10%, compared to another physiological state [11].

It has also been mentioned that females perform placentophagia to avoid attracting predators’ attention due to the volatile substances released by the placenta. Also, some great ape mothers have been observed climbing down from the tree’s safety canopy to the forest floor to retrieve placentas that fall from the nest after birth. In addition, many primate species are mobile immediately following parturition—both examples counter ‘predator avoidance’ placentophagia hypotheses. However, Menges [28] suggests that this may not be entirely true since it is observed that some animals, such as the tree squirrel, prefer to remove the placenta from the nest and throw it towards the ground instead of consuming it.

Other hypotheses attempting to explain placentophagia are related to the need to counteract nutritional losses due to pregnancy and delivery or to maintain the nest’s sanitary status [1,5].

## 3. Endocrine Effects

One of the causes of placentophagia may be related to the ingestion of hormones by females since the placenta contains hormones such as oxytocin, estrogens, progesterone, adrenocorticotropic hormone (ACTH), Releasing Factor Corticotropin [29], chorionic gonadotropin, hypothalamic releasing hormones (GnRH), placental lactogen, placental opioid enhancing factor (POEF), relaxin, and inhibin [30,31]. In females, the estrogens or similar sex hormones play a significant role in implanting the embryo and the development of the mammary gland. For its part, the amniotic fluid, contains hormones such as oxytocin, prostaglandins, androgens, renin, progesterone, corticosteroids, chorionic gonadotrophin, and placental lactogen [32]. It also has antibacterial activity and nutritional factors [32]. All these substances benefit the dam during the transition from pregnancy to the postpartum state and increase milk production.

Hammett and McNeile [20] were the first to publish that in humans, after postpartum ingestion of a dehydrated product derived from the placenta, an increase in protein and lactose was observed in breast milk. A subsequent study observed an increased growth rate in those children from mothers that ingested desiccated placenta capsules [33]. In the 1950s, Soyková-Pachnerová [34] confirmed the placenta’s capacity as a stimulator of lactation due to the hormone placental lactogen’s presence, among other components. Similarly, when cows can ingest the placenta and amniotic fluid, there is a marked increase in milk production [15].

The oxytocin present in the amniotic fluid and the placenta suggests that its ingestion may facilitate uterine contractility. Together with prostaglandins, it favors the cervix’s opening during the dilation process [32], allowing labor, delivery of the placenta, the cleaning of the uterus, and faster uterine involution. All this is assuming its absorption through the digestive tract and its action on maternal biology.

The role played during pregnancy by the different hypothalamic, pituitary, and gonadal hormones that the placenta contains has been extensively studied in both the mother and the fetus. However, in placentophagia, the effects that most hormones have on the mother are still under study since it has been found that the effect of hormones can exert different responses, depending on the animal species in which they are analyzed, as is the case of placental progesterone [35]. In this sense, Chabbert-Buffet et al. [35] found, by using sheep as a model, that altering progesterone levels causes dramatic changes in the GnRH pulse rate. Additionally, in the final stage of the follicular phase, progesterone plays a role in activating the Luteinizing Hormone (LH) surge in rodents, nonhuman primates, and women, but no such change is observed in sheep.

Most of the authors consider that in different animal species, the ingestion of hormones by placentophagia increases the mother and her offspring’s recognition and contact. This contact accelerates the emergence of maternal behavior. In contrast, in rats (*Rattus norvegicus*), Moltz et al. [36] observed that in females who underwent cesarean section and were later reintroduced to their clean pups, the bond between mother and pup developed normally.

A factor that has not been entirely studied is the level of digestive absorption of the different hormones present in the placenta. Therefore, there is no information on the endocrine effects that hormonal components may have on humans and nonhuman mammal species performing natural placentophagia. In this sense, the placenta’s preparation can modify the hormonal effect of placentophagia (fresh, raw, encapsulated, and processed). Therefore, minimal hormone levels have been found in processed human placenta capsules, which could hardly have endocrine effects in the body. However, some commercial placental preparations contain higher estradiol and progesterone concentrations, which could achieve high endocrine effects if the processed placenta’s consumption is high and due to the maximum concentration they contain. However, this requires further investigation and will depend on the recommendations of these product providers [29].

## 4. Analgesic Effects

The amniotic fluid and the placenta may act as morphinic inducers, facilitating analgesia and activating the release of endorphins [37,38] because the placental villi synthesize opioid peptides such as β-endorphin, metencephalin, and dynorphins. Ahmed et al. [39] found that dynorphin 1–8 is the main opioid peptide in placental extracts, associated with a substance also found in the placenta, called the placental opioid enhancing factor (POEF). POEF exerts an important influence in suppressing pain during parturition and triggering maternal behavior, producing changes in the central nervous system’s endogenous opioid activity [32]. This influence is due to increased delta and kappa receptors’ activity and mediators and a decrease in the mu receptors [40,41,42,43] (Table 1). It is essential to clarify that POEF is not an analgesic by itself [27] and does not potentiate analgesics such as aspirin [40] or nicotine. It is worth mentioning that the benefit to pregnant female mammals by ingesting amniotic fluid and placenta is that it improves opioid-mediated antinociception in the peripartum [44].

Abbott et al. [45] found that the POEF effect is generalizable to other species since it was observed in rats (*Rattus norvegicus*) that consume placental material from humans, dolphins, and cattle. It is also suggested that POEF is a product of the gastrointestinal system’s enzymatic or hydrochloric acid action. The rats injected with amniotic fluid subcutaneously and intraperitoneally did not show changes in morphine-mediated analgesia level compared to the rats administered orogastrically. Findings strongly suggest that POEF is a common mammalian substance, which suggests the possibility that all mammalian species, including humans, possess the capacity to respond to POEF. Currently, there are no controlled studies of the effects of POEF through placental consumption in humans [22,26,32].

The time to produce analgesia after ingesting placental material varies within species. In Holstein cows (*Bos taurus*) [41] and male rats (*Rattus norvegicus*) [45], analgesia occurs immediately and lasts up to 60 min; while in female rats (*Rattus norvegicus*), only a 30 min duration of the analgesic effect was registered [46]. Likewise, the dosage in the ingestion of placental material with an analgesic effect differs among mammal species. Kristal et al. [47] suggested that, in rats (*Rattus norvegicus*), the ideal amount of placental material to ingest was 0.25 mL of amniotic fluid and one placenta (500 mg), which are released at the birth of each pup. In contrast, Corpening [38] found that the effective dose of bovine amniotic fluid in rats (*Rattus norvegicus*) was 0.5 mL.

## 5. Nutritional Factors

Women may consume the placenta as an additional protein contribution in poor regions [48] since a single placenta weighing 450 g contains an average of 234 calories, 4 g fat, 899 mg cholesterol, 48 g protein, 513 g of sodium, plus significant amounts of trace elements such as iron, selenium, calcium, copper, magnesium, phosphorous, potassium, and zinc [22]. Placenta also contains essential and non-essential amino acids, such as alanine, aspartic acid, arginine, histidine, leucine, lysine, phenylalanine, proline, tyrosine, tryptophan, and valine, in addition to vitamins B1, B2, B5, B6, B7, B9, and B12 [22] (Table 1). Regarding Vitamin A and retinoids, the placental retinyl stores could be considered a local reserve for placental use. Transformation of retinyl esters into active retinol derivatives (RAs) could be a simple way to regulate in situ cellular proliferation and differentiation. This retinoid metabolism has been described in mouse and porcine placenta. Studies suggest that villous mesenchymal fibroblasts are primary sites of retinol esterification and storage in the placenta [61].

## 6. Benefits and Adverse Effects of Placentophagia

### 6.1. Benefits

Placentophagia in humans is currently only rarely practiced, possibly for cultural reasons [32]. There are currently no known, firsthand, ethnographic accounts of placentophagia among humans as a traditional cultural practice [62]. Interest in placentophagy has grown in industrialized cities in the last 50 years [22,29].

The placenta is most consumed through encapsulated supplements [63]. By ingesting it, mothers seek to obtain the supposed benefits attributed to this practice, such as increasing postpartum energy, improving mood, increase in milk production quality and quantity, improving weight gain of the newborn [29], as well as decreasing postpartum depression rates [64,65]. However, in contrast to this information, in a study by Young et al. [66], where Caucasian women were given placebo capsules and placental capsules, they found no significant differences in mood, level of fatigue, or the emotional bond between mother and newborn. Likewise, Gryder et al. [67] compared the effect of placental capsule consumption on postpartum iron levels against a beef placebo, in which they measured iron levels through blood samples four times: in the week 36 of pregnancy, within 96 h postpartum, between days 5 and 7 after delivery, and during the third week postpartum. The authors found that the iron concentrations in the placenta capsules were higher (0.664 mg/g) than the placebo capsules (0.093 mg/g), which was equivalent to only 24% of the recommended daily amount. However, no significant difference in maternal iron levels was found between women consuming placenta capsules or placebo. Therefore, it was concluded that the consumption of placental capsules does not improve postpartum maternal iron levels in women who eat an adequate diet; on the contrary, if the capsules are consumed as the only supplement, it can cause postpartum iron deficiency [67]. Other studies also report a lack of difference in the levels of milk production or the newborns’ weight gain [68], contrary to what Hammett and McNeile [19] reported.

Regarding the analgesic effect, POEF potentiates maternal behavior through opioid peptides in the ventral tegmental zone [37]. A study carried out on nonpregnant rats (*Rattus norvegicus*) observed that they show more interest in approaching newborns when they were bathed with placental material than when they were clean [69]. Conversely, Moltz et al. [36] found that rats’ maternal behavior was not significantly decreased when mothers receive their pups clean.

Other beneficial effects of placentophagia in animals are those reported by Blank and Friesen [52] in rats and include: the increase in the concentration of prolactin, decrease in the concentration of progesterone, and the increase in milk production. As mentioned before, an analgesic effect has also been observed as a result of the activity of the placental opioid enhancing factor (POEF) [40] (Table 1).

It is important to note that some factors determine the repercussions of placentophagia. At the experimental level, the effects of the placenta’s consumption by the mother depend on the mode of administration, time of intake after delivery, and dose. The mode of preparation and administration of placental tissues could affect the desired or purported beneficial elements. Kristal et al. [5] have shown in mice that the POEF effect is lost when the placenta is maintained at room temperature for more than 24 h. Hence, the best procedure is to freeze at −20 °C immediately after delivery and then heat it to 35–40 °C. An experiment conducted by Young et al. [29] showed that some nutrients were lost during the encapsulation process, so it is unlikely that the amount ingested will have relevant physiological effects or any clinical benefit. Similarly, Coyle et al. [26] mentioned that the way the capsules were processed could damage the fresh placenta’s beneficial molecular components. To counteract possible contaminating microbiological elements, the placenta for human consumption undergoes various conservation processes (dehydrated, cooked, or encapsulated). Unfortunately, at the end of such processing, the placenta loses many of the heat-labile substances considered beneficial [26,29] (Table 2).

The placenta may be ingested raw, cooked, or dehydrated (raw or steamed) and encapsulated into pills for use over time. The method of preparation varies depending on the service provider and the woman’s motivation for treatment. Some providers adhere to the Occupational Safety and Health Administration regulations to safely handle placental tissue. However, these regulations do not include evidence of the therapeutic efficacy of consumption. Other websites provide “do it yourself” instructions with an array of preparation methods, including baking placental tissue until “dry and crumbly” and placing it in a coffee grinder or food processer before encapsulating. However, these have not been standardized for efficacy or safety [26].

Interestingly, eating more than 3 or 4 placentas at intervals less than 30 min has been shown to have a reverse dose-effect [27,70]. The only human placentophagia Randomized Controlled Trial (RCT) to date utilized highly processed human placental tissue (steamed, high-temperature dehydrated, pulverized, and encapsulated).

On the other hand, Hayes [51] mentions that the hormones retained in the placenta, such as progesterone and estrogens, can provide specific beneficial postpartum effects, such as the relief of symptoms of depression and an increase in milk production (Table 1). However, Young et al. [29] analyzed the presence of 17 hormones in 28 placental samples that had been processed and discovered that only 15 of the 17 hormones analyzed existed in the encapsulated product. Hormones included cortisol, progesterone, androsterone, aldosterone, allopregnanolone, androstenedione, corticosterone, cortisone, testosterone, estradiol, estriol, estrone, melatonin, and testosterone. Furthermore, they found relatively low concentrations of these hormones, except for estradiol, progesterone, and allopregnanolone, that could have physiological effects due to the maximum concentration that some capsules contain (3300 mg/day). In contrast, melatonin and dihydrotestosterone were not found in all the samples or in lower concentrations than those detected by the method used [29].

### 6.2. Adverse Effects

The placenta may contain pollutants of a chemical or microbiological nature that can compromise the dam’s health if ingested since some toxic elements such as arsenic, lead, cadmium, and mercury have been found in placentas [71]. Although, these components are found in doses under toxic levels [22], and it cannot be verified that their consumption could cause poisoning (Table 2); however, this would depend on the case and could be at toxic levels.

An important factor is that the placenta is a tissue that has its own bacterial microbiota (Proteobacteria, Tenericutes, among others) [72] and can be easily contaminated; therefore, the form of its consumption influences its safety. Hence, the placental material consumption is contraindicated when the mother or newborn has a viral or bacterial disease. In humans, if the placenta is not cooked or prepared correctly, viruses such as Human Immunodeficiency Virus (HIV), hepatitis A, and Zika cannot be eliminated and might be a risk for those who ingest it [72] (Table 2). For example, the Hepatitis A virus requires temperatures above 70 °C for more than 4 min for inactivation, and the rotavirus for at least 30 min [73].

In dairy cows, their own or donor placenta’s consumption can transmit the agent *Neospora caninum*, a possible route of horizontal transmission and spread of this disease in the herd [74]. In cattle, colonization of the placenta by Brucella strains can cause abortion and excretion of large numbers of the agent at delivery. Thus, aborted fetuses, fetal membranes, and fluids contain large amounts of bacteria and pose a high risk of spread and infection if consumed or licked by other females [75,76] (Table 2).

Another adverse effect that limits the ingestion of the placenta in humans is the mother’s condition. If she has been administered general anesthesia during childbirth or cesarean section, its ingestion is not recommended due to anesthetic agents’ contamination. Similarly, when the mother is a smoker, the placenta could have cadmium deposits [22] (Table 2).

Due to the concentration of hormones identified, placentophagia can become an exogenous hormonal source and a health risk. Some authors have warned of the hypothetical risk of thromboembolism in women who ingest their placenta. Hayes [51] points out that there could be a risk of thromboembolism in women when consuming placenta due to estrogen’s presence in the placental tissue (Table 2). Thromboembolism from exposure to exogenous estrogens is rare, making it difficult to investigate without large numbers of study participants. To date, no such study has been conducted. Furthermore, there has been no case reports in the published literature of thromboembolism in a woman who consumed her placenta.

Stambough et al. [78] reported that maternal ingestion of the encapsulated placenta in a nursing mother became a source of exposure to exogenous estrogen for her baby, causing vaginal bleeding and breast budding at three months of age (Table 2). In other studies, Young et al. [68] evaluated the levels of prolactin after consuming the steamed, dehydrated, and encapsulated placenta in 12 women and compared them with the levels of 15 women who ingested placebos. The results obtained did not show statistical differences (*p* < 0.05), either in plasma prolactin concentration or in the newborns’ weight gain during the first three weeks postpartum.

It is worth mentioning that according to what was cited by Farr et al. [2], the Center for Disease Prevention and Control (CDC) of the United States recommends avoiding the intake of placental capsules because they can carry infectious pathogens via the encapsulation process itself. This is based on the case of a baby who presented with a B streptococcal infection associated with the fact that his mother consumed contaminated placenta capsules. The same bacterial strain was found in the baby’s blood analysis and the capsules [77] (Table 2). Furthermore, the Society of Obstetricians and Gynecologists of Canada does not recommend the practice of placentophagia due to the lack of safety involved in the process [79]. In contrast, Benyshek et al. [80], based on over 23,000 medical records, found no statistically significant differences in hospital admissions, neonatal intensive care unit admissions, or neonatal deaths among newborns of placentophagic (*n* = 7000) mothers versus non-placentophagic mothers (*n* = 16,000).

Theoretically, another disadvantage of placentophagia could be the possible risk of triggering alloimmunization, which can cause harm in future pregnancies. This condition is assumed to be caused by the recognition by T cells of paternal alloantigens contained in the placenta; however, this is entirely theoretical with no supporting data [2] (Table 2).

Young et al. [66] indicate that there is a great possibility that the benefits attributed to placentophagia, which have been reported by women who have practiced it, are more the result of a placebo effect than a scientifically validated and quantified effect. In a survey applied to women, approximately 1 in 5 of the interviewees mentioned that they practiced placentophagia because they believed that the proposed benefits outweighed the potential risks or were interested in alternative or natural health practices [21]. However, it does not mean that placentophagia does not benefit certain women after childbirth since the placebo effects are recognized for their therapeutic value in some contexts [66].

The Society of Obstetricians and Gynecologists of Canada does not recommend the practice of placentophagia because there are no scientifically validated studies. They indicate that these studies lack methodological scientific rigor to support the recommendations on the human placenta’s consumption [79].

## 7. Placentophagia in Veterinary Medicine

Placentophagia is a behavior that is commonly observed when dealing with mammals [54], especially females [1,5,6]. However, it is also observed in males of two-parent species [9,10,23] and some monogamous males who come to do it along with other paternal behaviors [12]. In addition to showing paternal behavior toward neonates, some males lick amniotic fluid before the birth, mechanically assist the delivery, open an airway by clearing the nostrils, lick and sniff pups after birth to clean the pups of membranes, and eat the placenta.

Some exceptions are marine mammals that give birth in the water, where expelled placenta’s chemical components are immediately diluted in the water [5,81].

Other exceptions have been noted in domestic animals (e.g., camelids—widely cited as an exception but for which there is little empirical evidence) whose behaviors are sometimes suspect because of selective breeding or captivity conditions [1,5,28,81].

As described above, there are many possible explanations for the phenomenon. However, each species presents differences in the behavior of placentophagia, possibly due to the species’ biological characteristics. That is, whether they are prey or predatory species (prey animals need to keep their nest clean to avoid attracting predators) or altricial or precocial animals (precocial species can be incorporated into group activities without needing to stay in a nest) (Table 1).

Placentophagy does not frequently occur in horses, and therefore it is not well documented. Confirmation of placentophagia in horses may have difficulties because the clinical implications in the detection of placental expulsion, management conditions of the neonatal foal, and also the potential for postpartum colic in the mare secondary to the placental ingestion, as an atypical substrate [82].

There is a positive correlation between the intake of amniotic fluid and consumption of the placenta, with the development of the bond between the mother and the offspring [16,54,55], observed in sheep [56], primates [58], bitches [54], and rabbits [59] (Table 1).

It should be noted that when a mutated version of the gene MEST1 is found in mice, maternal behavior and placentophagia is suppressed [83]. There is also a gene PEG3 analogous to MEST1, with the same effect in humans, but it is unknown if there is any homologous gene in other species [84].

### 7.1. Rodents

In some rodents, placentophagia is not exclusive to females; for example, male Djungarian hamsters (*Phodopus campbelli*) also consume the placenta and assist the female during their calving young. In addition to showing paternal behavior toward neonates, males licked amniotic fluid before the birth, mechanically assisted the delivery, opened an airway by clearing the nostrils, licked and sniffed pups in the moments and minutes after birth to clean the pups of membranes, and ate the placenta [10]. In hamsters (*Phodopus campbelli*), male and female offspring, 24 days old, who have remained in the nest together with their mother, also consume the placenta and amniotic fluid while the mother calves another litter [85]. Similarly, the male California monogamous mouse (*Peromyscus californicus*) also licked the female’s anogenital region, ate the placenta, and exhibited parental care as soon as the pups were born [9,23].

Abbott et al. [45] reported that, in both female and male rats (*Rattus norvegicus*), placentophagia behavior increased analgesia mediated by opioid peptides. Perea-Rodríguez et al. [9] found that ingestion of the placenta by the male California mouse (*Peromyscus californicus*) decreases anxiety behaviors but does not represent an influence on pain sensitivity, parental responses, or plasma corticosterone concentrations. Harding and Lonstein [11] found that weanling female laboratory rats participated in placentophagia while their mothers gave birth to a subsequent litter, and most ingested placenta outside the home cage.

Several studies suggest that the factor that stimulates placentophagia is related to oxytocin, which significantly increases when labor is near and immediately afterward triggers strong uterine contractions, as in rabbits [86]. Melo and González-Mariscal [12] tested estrous, midpregnant, prepartum (day 1), and postpartum (days 1 and 5) food-deprived rabbits with liver and placenta and evaluated the incidence of placentophagia in adult multiparous New Zealand rabbits (*Oryctolagus cuniculus*). They found that it occurs in 0%, 14%, 100%, and 50% during estrus, 8 h before farrowing, during labor, and one postpartum day, respectively. On the other hand, in rats (*Rattus norvegicus*), the incidence was 20% to 40% during estrus, 40–50% in prepartum, 100% during parturition, and 87% in postpartum [27]. In male mice (*Peromyscus californicus*), this behavior varies according to the reproductive stage in which it is found, being significantly higher when its mate is pregnant compared to when its mate is not pregnant [9].

### 7.2. Bitch

During a normal delivery, the dog licks and devours the placenta and fetal membranes, starting with cleaning the newborn puppy’s head, eating the placental remains, and biting the puppy’s umbilical cord [53,87,88,89], thereby reducing nest contamination and possibly preventing predators’ attraction [53]. This behavior of ingesting the placenta and its fluids is related to the dam’s acceptance of the offspring, the development of the mother–offspring bond, and the puppies’ survival, as demonstrated by Abitbol and Inglis [54]. They separated the newly whelped bitches from puppies at birth, cleaning the amniotic fluid and placental remains off the puppies. It was observed that, for the puppies that were resoiled with amniotic fluid, the bitches accepted the pups and generated a bond with them, while concerning the puppies who had their amniotic fluid wholly removed, the mothers rejected them (Table 1).

### 7.3. Cow and Buffalo

Newly calved cows are attracted to ingestion of the placenta and amniotic fluid, as reported by Machado et al. [15]. The latter conducted preference tests on the placenta and amniotic fluid consumption in Holstein cows during the peripartum. They conclude that cows prefer silages when mixed with donor cow placentas and that this attraction can occur even before calving.

In beef cattle, during licking of the calf and fetal membranes, cows frequently ingest some or all of the placenta 2 to 6 h after calving.

In buffalo, the time dedicated to this behavior is higher in the multiparous than in primiparous cows [90], similar to that observed in other ruminants [55].

In studies carried out by Pinheiro et al. [41], they observed that amniotic fluid consumption produced some analgesia for the cow and increased milk production [15]. The increase in analgesia observed in cattle after the ingestion of amniotic fluid immediately after calving lasted up to an hour after calving [27,32]. Also, in river buffalo (*Bubalus bubalis*), the amniotic fluid that covers the newborn attracts the mother, and licking the newborn helps to create the mother–calf bond [57] (Table 1).

### 7.4. Sheep and Goat

Placentophagia in animals called “hiders” such as small ruminants, has been studied in sheep (*Ovis aries*) and dairy goats (*Capra hircus*).

During the estrous cycle and practically all gestation, sheep have a strong aversion to amniotic fluid; in contrast, in the peripartum, these fluids become very attractive to the mother, reflected in an intense activity of licking the offspring by the mother [91], which initiates the ewe-lamb bond. However, there are few studies about placental consumption around the peripartum in sheep.

Regarding goats, Ramirez et al. [92] studied single and twin deliveries in the Murciano-Granadina breed and found that less than 20% of goats ignored the placenta. Furthermore, it was observed that no mother ingested the complete placenta; however, partial placentophagia was exhibited with a higher proportion in goats with twins (52%) than singletons (33.3%). Licking the placenta without consuming it was more frequent in goats delivering singletons (46.67%) than in mothers with twins (36%). Similar findings were observed by González-Stagnaro and Madrid-Bury [93] in primiparous and multiparous Creole goats in a tropical environment. Their research reported that primiparous goats (54.3%) showed more interest in the expelled placenta by sniffing, licking, and light grasping, without ingesting it, in comparison with multiparous goats (39%). However, less than 25% of the goats presented partial placentophagia (23.9% of primiparous and 17.1% of multiparous) (*p* < 0.05). On the contrary, in no case was complete placentophagia observed, regardless of the mothers’ parity.

### 7.5. Mare

Studies indicate that domestic horses, as well as camels or wildebeests, do not perform placentophagia. However, Virga and Houpt [82] surveyed horse owners and concluded that owners commonly remove the placenta (916/1425 (64.3%)). In some cases, before removing the placenta, the owners observed the mares investigating or examining the placenta (93/916 (10.2%)) or directly consuming the placenta (8/916 (0.9%)). Therefore, confirmation of placentophagia in horses may have difficulties because of the clinical implications in detecting placental expulsion because owners frequently remove the placenta [82].

One hypothesis as to why placentophagia is so rarely observed, could be because horses fit within the category of “followers”, or precocious species where the foals, after birth, move together with their mother from the place of birth. Therefore, mares are not required to ingest the placenta to protect against predators at the birth site [1]. Another hypothesis could be the “general hunger”, i.e., that anorexia before parturition leads to placentophagia as a means of maintaining homeostatic food intake requirements, and mares frequently show anorexia during labor [82].

## 8. Conclusions

Placentophagia is a common mammalian behavior that has gained popularity among women in the past 50 years for its purported benefits. Certain herbivorous mammals, such as the rabbit, buffalo, and cow, consume the placenta at the time of parturition, strengthening the mother–offspring bond. Also, this temporal change in their feeding habits could keep the nest clean and hidden from predators or because of the energy, hormones, and analgesic effect that its consumption contributes. Placentophagia is rare in species like horses or goats. In some rodent species, males may consume it.

In women, the controversy continues among researchers who report that there is no scientific rigor to support the benefits of placentophagia. In contrast, others attribute benefits to placentophagia, such as increased milk production, mood improvement, decreased postpartum depression, and fatigue, and more weight gain in the newborn. Still, other researchers claim that this practice might induce some health risks such as bacterial or viral infections or that the presence of trace elements could become toxic for both the mother and the baby. Likewise, the way the placenta is prepared, whether raw, cooked, dehydrated, or encapsulated, alters its components, and thus the desired effects might be lost. Placebo-controlled studies of placental capsules have not shown significant effects on hormonal levels, milk production, and weight gain in the newborn. Finally, in animal production systems, it is likely useful to allow animals to perform placentophagia since doing so reduces the risks of the mother rejecting the young in lactation. However, there are still questions to be resolved on the subject of placentophagia in animals and humans. More studies are needed to confirm or reject the data generated so far.

## Figures and Tables

**Table 1:** Potential advantages of placentophagia in women and nonhuman mammals.

Species	Condition	References
**Women and nonhuman mammals**		
Analgesia (Placental Opioid Enhancing Factor, POEF).	Amniotic fluid and placenta act as morphinic inducers, facilitating analgesia, and activating the release of endorphins	[37,38,39,40,41,42,43,45]
**Women**		
Postpartum benefits	Additional protein supplement in regions of extreme poverty. Source of trace elements and essential and non-essential amino acids and B vitamins	[48] [22]
Benefits for the newborn	Increases the concentration of lactose and protein in breast milk Increases the weight gain of the newborn	[20] [33]
Benefits for the mother	Improve mood after childbirth, convalescence is faster, and increases the amount of energy Relief of symptoms of depression and increase milk production	[2,22,26,49,50,51]
**Nonhuman mammals**		
Increase in milk production	Prolactin level increases, progesterone level decreases, and a possible increase in milk production	[52]
Protect from attack by predators	As there are no attractive chemicals, reduce nest contamination	[53]
Maternal behavior	It could accelerate the mother–calf bond in some species due to the oxytocin in the amniotic fluid or the placenta (sheep, primates, dog, rabbits, and river buffalo)	[16,54,55,56,57,58,59,60]

**Table 2:** Potential disadvantages of placentophagia in women and non-human mammals.

Species	Condition	References
**Women**		
Non-safe placenta preparations	Possible poisoning with arsenic, lead, cadmium, and mercury Possible infection with viruses (HIV, hepatitis A, and Zika) Contamination with *Streptococcus* type B With anesthetic residues or metabolites Cadmium deposits in smoking mothers	[22,71,72,77]
Placenta processed	Lose active substances that are considered beneficial	[26,29]
An exogenous hormonal source with health risk	Extra contribution of exogenous estrogens in breast milk induced vaginal bleeding and mammary budding in a 3-month-old baby. Thromboembolism in women	[78] [51]
Possible alloimmunization in future pregnancies	T-cell recognition of paternal alloantigens contained in the placenta	[2]
**Nonhuman mammals**		
Dissemination of diseases in the herd	Horizontal transmission of *Neospora caninum* (cows)	[74]
Risks of dissemination of diseases in cattle herds	Dissemination and transmission of *Brucella* strains in cows due to the consumption of placentas and contaminated fluids	[75,76]
